# Prevalence of glue‐sniffing among street children

**DOI:** 10.1002/nop2.380

**Published:** 2019-09-30

**Authors:** Sanjay Kumar Sah, Nira Neupane, Anupama Pradhan (Thaiba), Sabita Shah, Asha Sharma

**Affiliations:** ^1^ Chongqing Medical University Chongqing China; ^2^ Sanjeevani College of Medical Sciences Butwal Nepal

**Keywords:** glue, nurses, nursing, prevalence, sniffing, street children

## Abstract

**Aim:**

The aim was to determine the prevalence of glue‐sniffing among street children.

**Design:**

A descriptive cross‐sectional study design was adopted.

**Background:**

Children are the source of hope and could be the major ailments for the development of society, nation and the world but there are large numbers of children on the street sniff glue and struggling with multiple disease and uncertain future.

**Methods:**

A study was conducted in 52 street children to determine the prevalence of glue‐sniffers and its impact on street children in Butwal, municipality of Nepal. This study was conducted in 52 street children, 5‐ point Likert scale and interview schedule was performed with the help of structured and semi‐structured questionnaire to collect data. Finally, the collected data are analysed by using descriptive statistical methods such as frequency, percentage and mean.

**Results:**

The study findings revealed that a large number of children, that is 40.38% was between the age group of 9–12 years and 92.31% were male. The current prevalence of glue‐sniffing among street children is 88.46%. Similarly, 58.7% of respondents had started glue‐sniffing 1 year ago. Out of 46 respondents who sniffed glue, 89.13% were unaware of its effect. Nearly, half of the respondents 45.65% had encountered health issues such as headache, chest pain and stomach ache.

**Conclusions:**

They have deprived children and denied not only of their rights as children but also of their normal childhood. Without guidance, education and security, they are heading towards an uncertain future. They can have enough potentiality and talent if they are brought into a better environment and might have real hope for the bright future.

## 1 INTRODUCTION

During the early 1960s, the effect of inhaling substances and their impacts on the society and users health become major issue to the researcher and government as well. Meanwhile, it is also reported about the abuse of other kinds of volatile substances, such as paint thinners, nail varnish remover, gasoline and lighter fluids subsequently appeared. However, the term “glue‐sniffing” has come to be applied, in a general way, to all kinds of solvent abuse by inhalation. Glue‐sniffing is usually taken by the young children as a forerunner to alcohol abuse, and probably, it became substitute because of its low cost, readily availability and provides faster onset of action (Barker & Adams, 1962).

Children's right has become a serious agenda of the developing world, according to United Nations charter on the rights of the child in 1990; street children in the large cities of the world received much attention to provide their quality life and better future (Bourdillon, 1994).Children are the source of hope, stimulus for the society and could be the building blocks for the development of world so they should not remain neglected by parents or government rather than bringing them into positive environment. Though, there are many children found working or living on the street of urban area throughout the world. Populations of Street children are marginalized mainly in urban area and look quite difficult to trace the exact number and the magnitude of their hardship. In their marginalized state, they constitute a truly “hidden” population who were excluded from the national census, educational or health data, largely because of lacking of their proper address. (Nandan Kumar Mondal, 2013)

There is study suggested that children who starts abusing inhalants or other solvents in early stages of life they are more likely later to use other illicit drugs in their future. Thus, inhalant abuse intervention programmes seem important from the community health perspective also (Mondal, 2013).

Hence, just like abuse of any other substances sniffing is a serious problem among street children. The main cities of Nepal where the street children found are Kathmandu, Biratnagar, Pokhara, Dharan, Narayanghat and Butwal. According to a prominent child rights NGO, Child Workers in Nepal (CWIN), there are approximately 5,000 street children over the country among them 800–900 in Kathmandu only. Glue‐sniffing among street children in Nepal can be taken as an initiation to the use of other more teratogenic drugs. Peer pressure and easy availability of glue “dendrite” are two of the prominent reasons for addiction to sniffing. Addiction to glue‐sniffing is making street children more dependent on substances. Such type of drug dependency is resulting in an increase in street children carrying out petty thefts. This trend, in the course of time will lead children to even more violent and unsocial activities (Singh, Tripathi, Karki, & Bhandari, 2009).

## 2 BACKGROUND

Glue sniffing is discovered as comprehensive preponderating as an inhalant use. In USA approximately 20% of pubescent have speculated its application (Kurtzman, 2001). The average age of initial inhalant abusers is discovered 13 years with the onslaught in children at 6 to 8 years. in addition, a repudiate in its use occurs at the age of 17 to 19 yrs (McGarvey, 1999). Nevertheless, some abusers persist the addiction up to adultness. Again, other felonious medicines are discovered as forerunner of it in later years (Bennett, 2000) (Tulsidas, 2010)

Many heroin addicts and IV drug abusers have a past history of inhalant use, which is more common in those from a lower socioeconomic background, particularly those from chaotic, broken homes and abusive families. Other risk factors for inhalant abuse include those with aggressive behaviour, low self esteem, positive family history of abuse or alcoholism, peer pressure, poor academic achievement, abuse or neglect and those who have been exposed to violence or assault. Heavy inhalant abusers, especially females, are more likely to have had a background of childhood abuse, either physical or sexual, than non‐abusers or lighter abusers (Tulsidas, 2010).

A survey was conducted in Dhaka and Chittagong by selecting sample using random sampling technique among 640 street children aged between 11–19 years. Most respondents was slum dwellers with very limited educational attainments and involved in low income‐generating activities, mostly rag picking and begging. Lack of food appeared to be the most compelling reasons for the respondents to be on the streets with almost three‐quarter of the respondents identifying these factors as the main causes. Other reasons included seeking employment, avoiding or overcoming domestic violence and the influence of peer. Abuse (both physical and verbal) was the biggest problem faced by the street children, mostly caused by police and peers. Adhesive was the most common solvent used by the street children along with thinners and balm. In addition to glue and cannabis, cigarette smoking was also found to be pervasive among the respondents. The reasons stated for glue‐sniffing included peer pressure, curiosity, pleasure, coping with stress and overcoming family issues. Respondents who slept in public places and parks/ streets were more likely to sniff glue—25% of the glue‐sniffers slept in parks/ streets compared with 13% non‐glue‐sniffers; 28% of the glue‐sniffers slept in other public places compared with 14% of the non‐glue‐sniffers. Moreover, street children who sniffed glue were more likely to use other drugs than non‐glue‐sniffers (Mahmud, Zunaid, & Claeson, 2011).

A research conducted in Pakistan in a total number of 416 children with equal distribution from all four cities, that is Karachi, Lahore, Peshawar and Quetta participated in the study. Respondents were predominantly males, with only 07 girls (1.7%) included in the sample. The mean age of children using solvents was 14.3 (*SD* 2.52) years, with the children in Peshawar slightly younger (13.0 *SD* 2.33 years) than the children found in other cities. The maximum proportions of children were between 15–16 years of age. The largest proportions of children interviewed were Pakistani (81%), more than half of whom had migrated from smaller cities. 12.5% of the total children were Afghani followed by Bengalis (5.8%). One third of the children interviewed belonged to single‐parent families with large family size. Almost three quarters of the children were not currently living along with their families. These children prefer staying in groups, had no permanent place to live, had been sleeping in parks (50%), friend's place (12%) and darbars (6.7%) etc. (76%) of the children never attended formal education. The remaining 24% had 3.01 (*SD* 1.97) years of educational attainment. The average daily income reported was Rs. 79.7 (*SD* 44.7), which was sourced through cleaning cars, scavenging solid waste garbage and begging (UN‐PAK/UNODC/, 2004).

Of the children 55% complained of at least one medical problem that they were currently facing. The major problem reported was Respiratory tract infection (30%), followed by fever (19.7%), GIT upsets (18.7%) and skin infections (12.6%). Adhesive glue was the primary drug of choice consumed by 374 (90%) of the interviewed street children. Other solvents abused included petrol (25.5%) and thinners (10.6%). Use of petrol was significantly popular in Quetta (43%) and Peshawar (41%). In Karachi, children were found to be involved with glue (95.2%), thinners (27.6%), petrol (13.3%), as well as tincture (5.7%). An average amount of Rs. 39.7 ± 30.1 was being spent on these solvents with minimal geographical variations. A little more than 60% of the children reported regular use of these substances for more than 2 years via various techniques. On average they inhaled almost 80 g (UN‐PAK/UNODC/, 2004).

A cross‐sectional descriptive study showed that 68.8% of the street children were between 11–15 years of age and in overall 95.8% were males. Out of the total participants, 81.2% were found to be rag pickers. Research findings revealed that 100% of the participants had at least one or more health problems. From the study it was discovered that most 87.5% had the habit of cigarette smoking, 50% had habit of consuming alcohol and 72.9% had the habit of taking drugs. Dendrite (glue‐sniffing) was the only drug used by the respondents in this study. The most common health problems faced by them were head lice infestation (81.2%), headache (66.7%), cut injury (60.4%), common cold (52.1%), dental caries (52%), burning micturation (47.9%), cough (47.9%), being underweight (43.8%), abdominal pain (39.6%), tinnitus (37.55%), gum bleeding (33.3%), joint pain (31.2%), eye inflammation (25%), leg cramps (25%), palpable lymph nodes (25%), chest pain (18.8%), skin lesions (16.7%) and abnormal vision (8.3%) (Thapa, Ghatane, & Rimal, 2009).

The reason for conducting this study describes that there are many children on the streets of western Nepal and involved in unsocial activities like glue‐sniffing, drug addiction, violence, stealing and pickpocketing, but no studies have been found. There are numerous studies conducted on the glue‐sniffing and uses of inhalants worldwide but only few studies have been conducted in Nepal despite there being many street children involved in glue‐sniffing and putting them in danger.

### 2.1 Aim

The researcher aimed to assess the prevalence of glue‐sniffers and its impact in street children of Butwal municipality, Nepal.

## 3 DESIGN/METHOD

### 3.1 Research design

A descriptive cross‐sectional study design was adopted to accomplish this study.

### 3.2 Setting

The data for main study was collected from three wards of Butwal Municipality among street children between 5–17 years old, from 19 December 2016–18 February 2017.

The reliability was established by pre‐testing the research instrument in 10% of the total population similar to sample population in ward no. 12 of Butwal Municipality, located in the western part of Nepal, 300 km south‐west of capital city Kathmandu. The Butwal city is one of the fastest growing municipalities in western Nepal with a population of 25–2686 in 2018.

### 3.3 Participants

The study participants were street children who were working or living on the road to sustain their life by collecting the waste or begging. The sample consisted of 52 participants selected by non‐probability purposive sampling technique. Preparation of the tool was done on the basis of literature review, content validation and establishment of reliability. The content validity was established by consulting various literatures, peer review and subject experts.

### 3.4 Instruments

This study is divided into two sections: section first is related to distribution of participants based on demographic variables and section second about questionnaire of glue‐sniffing. An extensive literature search was carried out to get an idea on the structure of knowledge regarding questionnaire about prevalence of glue‐sniffing among street children. The questionnaire was modified in such a way that it fitted to involve the many activities of street children in terms of using glue or other kinds of inhalants or compounds. It consisted of 11 items with some items having multiple responses. After finalizing the structure of the tools, intensive group discussions were conducted, and questionnaire was decided into final form on the basis of expert’s opinions.

**Table 1:** Frequency and percentage regarding prevalence of glue‐sniffing among street children

Variables	Frequency (*f*)	Percentage (%)
Glue‐sniffing among street children (*N* = 52)		
Yes	46	88.46
No	6	11.54
Duration of beginning glue‐sniffing (*N* = 46)		
2 years	9	19.57
1 year	27	58.7
6 months ago	7	15.22
Others	3	6.52
Sniffing glue per day (*N* = 46)		
Once	1	2.17
2–5 times	13	28.26
More than five times	25	54.35
Other (One tube per day)	7	15.22
Place for buying glue (*N* = 46)		
Shops	40	87
Friends	6	13
Cobbler	–	–
Others	–	–
Group with whom children used to sniff glue (*N* = 46)		
Friends	34	73.91
Bullies	9	19.57
Alone	3	6.52
Others	–	–
Reasons for coming on street (*N* = 52)		
Peer pressure	11	21.15
Lack of food	4	7.7
Seeking employment	37	71.15
Others	–	–
Reasons behind glue‐sniffing (*N* = 46)		
To cope with stress	24	52.17
For becoming mentally strong	13	28.26
To cope with hunger	9	19.57
For pleasure	–	–
Awareness among street children towards effect of glue‐sniffing (*N* = 46)		
Yes	5	10.87
No	41	89.13
Different types of substances used by street children (*N* = 52)*		
Alcohol	46	95.83
Tobacco	28	58.33
Cigarette	35	72.92
Glue/Dendrite	46	95.83
Gutkha	44	91.67
Health problems encountered during glue‐sniffing (*N* = 46)		
Headache/Nausea	3	6.52
Chest pain	7	15.22
Stomach ache	11	23.91
Others (not encountered)	25	54.35
Other issues apart from health problems among street children (*N* = 52)		
Yes	45	86.54
No	7	13.46

* Multiple responses.

### 3.5 Data analysis

The collected data were checked, reviewed and organized for accuracy and completeness. Editing and coding of the data were carried out. All the data were entered in Microsoft excel 2007. The obtained data were analysed by using descriptive statistics. The analysis was based on the objectives of the study. Frequency and percentage were used to analyse the baseline Performa.

The objective of this study were to identify socio‐demographic data of the respondents, to discover the pattern and frequency of glue‐sniffing, to find out the reasons behind glue‐sniffing and to determine the effects of glue‐sniffing on street children. The data were presented under the participants based on demographic variables and prevalence of glue‐sniffing among street children.

## 4 RESULTS

The analysis and interpretation of data was done to find out the prevalence of glue‐sniffing among street children. Analysis of data is a process by which quantitative information is reduced, organized, summarized, evaluated, interpreted and communicated in a meaningful way. The results were computed by using descriptive statistics based on the objectives of the study.

### 4.1 Distribution of participants based on demographic variables

**Figure 1:**
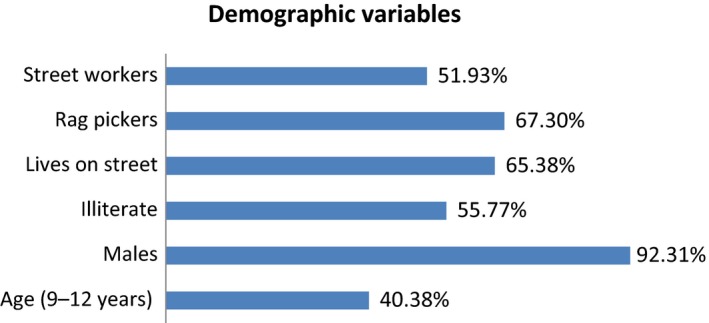
Demographic variables

Out of total samples (*N* = 52), most of the respondents (40.38%) were of age group 9–12 years mean (mean age was 10.07) where 92.31% were males whereas only 7.69% females. Participants based on education showed that maximum of the respondents 55.77% were illiterate; however, 65.38% respondents lived on the street (Figure 1). Most of the respondents 67.3% picked the rag for their livelihood and 51.92% had been working on the street since more than 1 year, 5.77% and 26.92% respondents were found port and begging on the street, respectively. 34.62% respondents had been working on the street between 6 months–1 year, 9.62% respondents had been doing so since 3–6 months and 3.85% had been recently working since less than 3 months (Figure 1).

### 4.2 Findings regarding prevalence of glue‐sniffing among street children

The statistical analysis of present study showed that the prevalence of glue‐sniffing is 88.46%. Most respondents (40.38%) were 9–12 years old. Maximum respondents (92.31%) were boys, 55.77% respondents were illiterate, only 34.62% respondents lived with their family and most of them (67.31%) used to pick rags for their livelihood, 26.92% children begged in different places of Butwal. 51.92% children had been living on street for more than 1 year.

Among the 46 glue‐sniffers, maximum (58.7%) started glue‐sniffing since 1 year and 25 (54.35%) of them were sniffing glue more than five times a day. Similarly, most of them (87%) brought glue from shops and most respondents (73.91%) used to sniff glue with their friends. Likewise, most of the respondents (71.5%) came on the street to seek employment and major reason behind the glue‐sniffing was to cope with stress. Among total glue‐sniffers, 89.13% did not know about the effect of glue‐sniffing. Only 10.87% possessed the knowledge regarding effect of glue‐sniffing. Along with glue, street children used to abuse different types of substances such as alcohol, tobacco, cigarette and gutkha (tobacco with betel nut). In the same way, most of them (54.35%) told that they had not encountered any kind of health problems. 23.91% complained about stomach ache, 15.22% and 6.52% of them had suffered from chest pain and headache/nausea respectively. Out of total respondents, 86.54% had faced other various kinds of problems apart from those related to health like arrested by police, beaten and scolded by people, frostbite and rain.

Out of 52 respondents, 46 respondents sniffed glue. Therefore, the prevalence rate of glue‐sniffing in ward no. 4, 6 and 13 of Butwal Municipality was found to be 88.46% (Table 1).

## 5 DISCUSSION

The present study showed that the prevalence of glue‐sniffing was 88.46% comprising of 92.31% male and participant age were from 9–12 years. This finding is very close to one conducted by the *Kathmandu University Medical Journal* in 2009 showing that 68.8% of the street children were between 11–15 years of age, 95.8% were males. Also, out of the total respondents, 81.2% were found to be rag pickers but in our study, it was only 67.31%. All of the participants had at least one or more health problems whereas present study suggested only 86.54%. Furthermore, present study implicated 72% were involved in cigarette smoking and 95% alcoholic while 87.5% had the habit of cigarette smoking and 50% of consuming alcohol Thapa et al., 2009).

The findings by Gebrehiwet et al showed that more than 95% of the children had families, about 40% of the children lived with their families and 55% were living on the street; however, present study showed that most respondents were unaware of their parents and family members, only 34.62% lived with families and 65.38% are living on the street. Present study also found that domestic violence, seeking employment, poverty, abuse and deprivation from education were the main reasons that caused them to leave their families which were not investigated by previous study. More than 70% of them preferred to sniff in a group but we noted only 34% sniffed in a group. This difference in results may have occurred because of their loneliness nature and they always found walking through the city in terms of searching the waste goods to generate some income (Gebrehiwet, Yimesghen, & Metwally, 2014).

A total of 118 street children with the age 10–16 years from different areas in Kathmandu were interviewed and found that only 43% street children were using glue in different quantity which was very low in comparison to the present study. After arriving on the streets, they reported of taking up all kinds of works like begging, rag picking, tempo conducting, porting, stealing and boot polishing. This difference in the findings may be because of they were more concentrated on their work at different places like junkyards, temples, markets, cinemas, the airport, bus terminals, hardware shops and tourist centres. In addition, it also suggests that children were actively involved in using any kind's solvents or drugs when they were on groups (Rai, Ghimire, Shrestha, & Tuladhar, 2002).

### 5.1 Limitations

In this study, data were collected from only certain ward of Butwal city of western Nepal which limits the external validity of the result.

## 6 CONCLUSION

Street living has become a common phenomenon for children in many cities of Nepal. Children who are working and living on the streets are found throughout the nation. The definition of “street” is no longer limited to being a path but is now interpreted as a site for living for many children in our country. Street children are among the high risk and insecure groups and are consequently vulnerable to various forms of exploitation and abuse. They are deprived children, denied not only of their rights as children but also of their childhood. Without guidance, education and security, they are heading towards an uncertain future. They have enough potentiality and talent. If they are brought into better environment, they could have a real hope for the future.

## CONFLICT OF INTEREST

There is no conflict of interest in this study.

## AUTHOR CONTRIBUTIONS

Sah SK: Contribution in idea and conception of study. NN: Contribution in formation of questionnaire and online database search. SS: Editing and revising of the whole article. AP and AS: Collection of data. All the authors have read carefully and approved the final manuscript.

## ETHICAL APPROVAL

This study was not required the ethical clearance from the institutional review committee (IRC).

## RECOMMENDATIONS

On the basis of finding, it is recommended that street children are vulnerable to many things including drug abuse and health complications. Programme with broad overview of street children's issues is needed to prevent from getting into substance addiction and drug abuse. The government of Nepal should prohibit the sale of glue and other volatile solvent agents to children. Also, shopkeepers should be made aware about the ill effects of glue on children and should be punished for selling such substances to children. Additionally, further research is required with large sample size to see the prevalence of glue‐sniffing and its complications among adolescents in various major cities of country.

7

**Figure 2:**
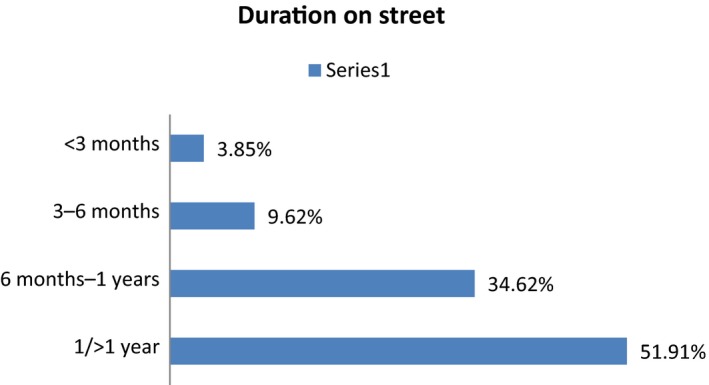
Duration on the street
